# MASTL(Greatwall) regulates DNA damage responses by coordinating mitotic entry after checkpoint recovery and APC/C activation

**DOI:** 10.1038/srep22230

**Published:** 2016-02-29

**Authors:** Po Yee Wong, Hoi Tang Ma, Hyun-jung Lee, Randy Y. C. Poon

**Affiliations:** 1 Division of Life Science, Center for Cancer Research, and State Key Laboratory of Molecular Neuroscience, Hong Kong University of Science and Technology, Clear Water Bay, Hong Kong

## Abstract

The G_2_ DNA damage checkpoint is one of the most important mechanisms controlling G_2_–mitosis transition. The kinase Greatwall (MASTL in human) promotes normal G_2_–mitosis transition by inhibiting PP2A via ARPP19 and ENSA. In this study, we demonstrate that MASTL is critical for maintaining genome integrity after DNA damage. Although MASTL did not affect the activation of DNA damage responses and subsequent repair, it determined the timing of entry into mitosis and the subsequent fate of the recovering cells. Constitutively active MASTL promoted dephosphorylation of CDK1^Tyr15^ and accelerated mitotic entry after DNA damage. Conversely, downregulation of MASTL or ARPP19/ENSA delayed mitotic entry. Remarkably, APC/C was activated precociously, resulting in the damaged cells progressing from G_2_ directly to G_1_ and skipping mitosis all together. Collectively, these results established that precise control of MASTL is essential to couple DNA damage to mitosis through the rate of mitotic entry and APC/C activation.

Greatwall kinase (MASTL in human) has emerged as a key player for mitosis. Greatwall/MASTL phosphorylates two small proteins called ARPP19 and α-endosulfine (ENSA), stimulating their inhibition of PP2A–B55δ^1^,^2^. As PP2A–B55δ is the major phosphatase that dephosphorylates CDK1 substrates, Greatwall/MASTL plays a pivotal role in maintaining CDK1-dependent phosphorylation during mitosis^3^,^4^,^5^ CDK1 activates Greatwall/MASTL during mitosis in a positive feedback loop^6^.

A crucial role of Greatwall/MASTL appears to be for regulating the activity of cyclin B1–CDK1. In support of this, depletion of Greatwall in *Xenopus* egg extracts stimulates the accumulation of Thr14/Tyr15-phosphorylated CDK1^6^, which could in part be explained by the maintenance of CDC25 and WEE1 phosphorylation by the Greatwall/MASTL pathway^7^. In human cells, depletion of MASTL also induces a G_2_ arrest. Partial depletion of MASTL, however, induces multiple mitotic defects that include the spindle-assembly checkpoint and cytokinesis, indicating that MASTL is also important for the maintenance of cyclin B1–CDK1 activity during mitosis^3^. In agreement with this, conditional knockout of the mouse MASTL indicates that cells can enter mitosis with normal kinetics without MASTL, but they display mitotic collapse after nuclear envelope breakdown^8^.

To maintain genome integrity, it is vital for cells to halt mitotic entry after DNA damage. A surveillance mechanism termed the G_2_ DNA damage checkpoint monitors DNA integrity and prevents entry into mitosis^9^. Following DNA double-strand breaks, ATM is autophosphorylated, leading to release of active monomers from homodimer complexes. ATM then phosphorylates residues in the SQ/TQ domain of CHK1/CHK2, stimulating the activity of these effector kinases^10^. CHK1/CHK2 in turn activates WEE1 and represses the CDC25 phosphatase family, thereby maintaining CDK1 in an inhibitory phosphorylated state^11^.

Given that MASTL is now established as a key regulator of G_2_ and mitosis, we hypothesize that MASTL may play a role in preventing damaged cells from entering mitosis. Indeed, there is evidence from experiments using *Xenopus* egg extracts that Greatwall is inhibited after DNA damage and its activity is required for checkpoint recovery^12^. Furthermore, direct association and phosphorylation by Plx1 (PLK1 homolog in *Xenopus*) are important for the function of Greatwall in checkpoint recovery^13^. Whether this is true for DNA damage responses in the cellular context remains an important and outstanding issue. Here we found that in human cells, MASTL determines the timing of mitotic entry after DNA damage and provides a link between the G_2_ DNA damage checkpoint and APC/C activity.

## Results

### The rapid G_2_ DNA damage responses are not dependent on MASTL

**Figure 1:**
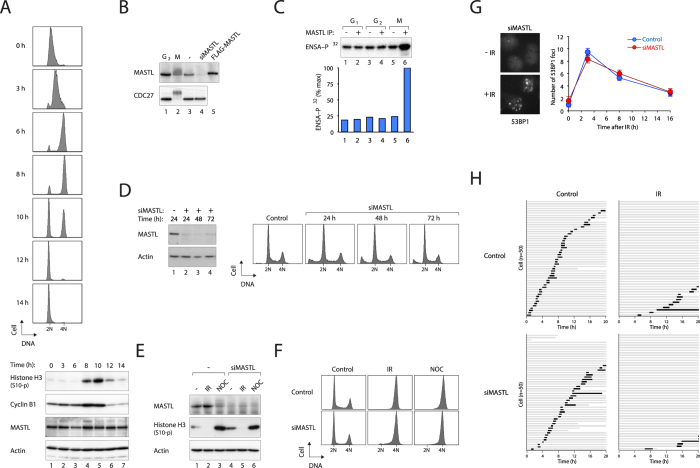
Activation of the G_2_ DNA damage checkpoint is independent on MASTL. (**A**) MASTL does not vary significantly during the cell cycle. HeLa cells were synchronized using a double thymidine procedure. At different time points, the cells were harvested and analyzed with flow cytometry (upper panel) and immunoblotting (lower panel). Cyclin B1 and histone H3^Ser10^ phosphorylation indicated the timing of mitosis. (**B**) MASTL in G_2_ and mitosis. Lysates from G_2_ and mitosis (M) were analyzed with immunoblotting. MASTL was extensively phosphorylated during mitosis. Cells transfected with MASTL siRNA (siMASTL) or plasmids expressing FLAG-MASTL were loaded to validate the identity of the MASTL band. (**C**) Low kinase activity of MASTL during interphase. Different lysates were subjected to immunoprecipitation using either control or MASTL antiserum. The kinase activity was assayed using purified 6xHis-ENSA as a substrate. (**D**) Downregulation of MASTL does not affect unperturbed cell cycle distribution. Transfected HeLa cells were harvested at the indicated time points and analyzed. (**E**) IR-mediated interphase arrest is independent on MASTL. HeLa cells were transfected as indicated. After 48 h, the cells were treated with IR (15 Gy) or incubated with nocodazole (NOC). After another 16 h, the cells were harvested for immunoblotting. (**F**) IR-induced G_2_ arrest is independent on MASTL. HeLa cells transfected as indicated were irradiated (15 Gy). After another 16 h, the cells were analyzed with flow cytometry. NOC treatment (16 h) was used as a mitotic control. (**G**) MASTL does not affect IR-mediated 53BP1 foci formation or repair. HeLa cells transfected as indicated were irradiated (2 Gy) and fixed at different time points. The number of 53BP1 foci per cell was quantified (*n* = 100; mean ± 95% CI). Examples of 53BP1 staining of siMASTL-transfected cells in the presence and absence of IR treatment (3 h) are shown. (**H**) Mitosis is inhibited by IR in the absence of MASTL. HeLa cells expressing histone H2B-GFP were transfected as indicated. After 48 h, the cells were either mock-treated or irradiated (15 Gy). Individual cells were then tracked with time-lapse microscopy. Each horizontal bar represents one cell (*n* = 50). Grey: interphase; black: mitosis (from DNA condensation to anaphase); truncated bars: cell death.

We first used synchronized HeLa cells to verify that the abundance of MASTL remained constant during G_2_ and mitosis (Fig. 1A). Bacterially expressed ENSA was used as a substrate to assay the kinase activity of the MASTL. Coincided with the gel mobility shifts of MASTL (Fig. 1B), the kinase activity of MASTL from mitotic cells was higher than that of MASTL isolated from G_1_ or G_2_ (Fig. 1C).

As MASTL is normally activated during mitosis, we hypothesized that keeping MASTL inactive is important for the proper function of the G_2_ DNA damage checkpoint. To test this hypothesis, we initially examined if the activation of the checkpoint is compromised when MASTL was either overexpressed or depleted. HeLa cells were used as a model in this study because IR exclusively activated the G_2_ DNA damage checkpoint in these cells (p53 is degraded by HPV E6).

Downregulation of MASTL with siRNA (siMASTL) did not affect unperturbed cell cycle distribution (Fig. 1D). Figure 1E shows that the expression of MASTL was not altered by ionizing radiation (IR)-mediated DNA damage. Inhibition of mitotic entry (which is reflected by the absence of histone H3^Ser10^ phosphorylation) was not affected by MASTL depletion. Both control and MASTL-depleted cells were arrested with G_2_/M DNA contents at 16 hours after irradiation (Fig. 1F). Expression of siMASTL also did not alter the number of IR-induced 53BP1 foci, indicating that the early responses to double strand breaks were unaffected by MASTL (Fig. 1G). We further validated the presence of G_2_ DNA damage responses by using live-cell imaging to follow individual cells after DNA damage. Figure 1H shows that although transfection of siMASTL extended the interphase in the unperturbed cell cycle, it did not abolish mitotic entry. Treatment with IR inhibited mitotic entry in both control and siMASTL-transfected cells, indicating that downregulation of MASTL did not compromise the DNA damage-mediated G_2_ delay.

**Figure 2:**
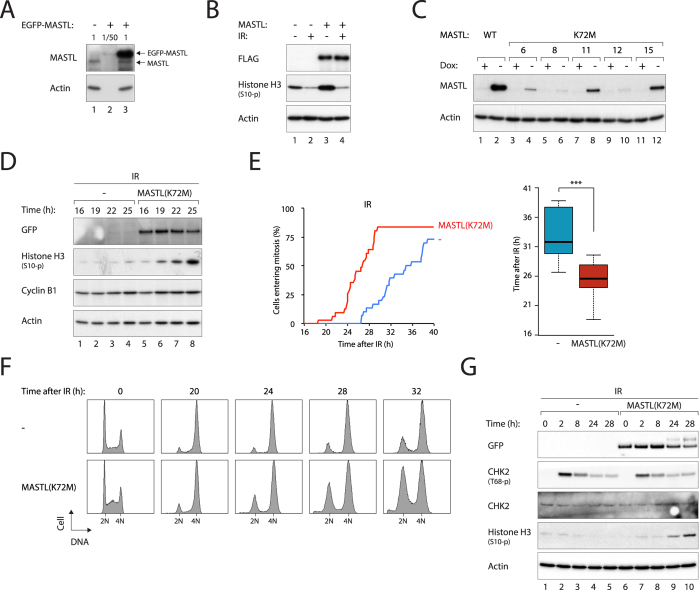
DNA damage-induced G2 arrest can be interrupted by MASTL. (**A**) Ectopic expression of MASTL. A HeLa cell line expressing FLAG-EGFP-MASTL was generated. Lysates were prepared and different dilutions were loaded to compare with normal HeLa cells. (**B**) Overexpression of MASTL does not affect checkpoint activation. HeLa cells expressing FLAG-EGFP-MASTL were irradiated (15 Gy). NOC was added to trap cells in mitosis. After 6 h, the cells were harvested and analyzed. The expression of exogenous MASTL was detected with antibodies against FLAG. (**C**) Expression of MASTL^K72M^. Cell lines expressing FLAG-EGFP-MASTL^K72M^ under the control of doxycycline (Dox) were generated. MASTL^K72M^ expression in different clones was compared to the cell line expressing wild type MASTL. Clone#15 was used in other experiments in this paper. (**D**) MASTL^K72M^ promotes mitotic entry after DNA damage. Cells were grown in the absence or presence of Dox for 48 h to turn on or off MASTL^K72M^ respectively before treated with IR (15 Gy). After 16 h, the cells were incubated with NOC and harvested at different time points for immunoblotting. (**E**) MASTL^K72M^ accelerates mitotic entry after DNA damage. Cells were grown in the absence or presence of Dox for 48 h. The cells were then transfected with a plasmid expressing histone H2B-mRFP. After 24 h, the cells were treated with IR (15 Gy). After another 16 h, individual cells were tracked with time-lapse microscopy to analyze the time of mitotic entry (*n* = 50). The median mitotic entry time was plotted as a box-and-whisker chart. Expression of MASTL^K72M^ significantly accelerated entry into mitosis (*P *< 0.001). (**F**) MASTL^K72M^ shortens G_2_ arrest after DNA damage. Cells were grown in the absence or presence of Dox for 48 h. After treating with IR (15 Gy), the cells were harvested at the indicated time points and analyzed with flow cytometry. (**G**) MASTL^K72M^ does not affect inactivation of the checkpoint. Cells were grown in the absence or presence of Dox for 48 h. After treating with IR (15 Gy) and NOC, the cells were harvested at the indicated time points. The expression of the indicated proteins was detected with immunoblotting.

To determine if overexpression of MASTL affects the DNA damage responses, we generated cell lines expressing MASTL under the control of doxycycline (Dox). The recombinant MASTL could be distinguished from the endogenous MASTL by the change in size caused by the FLAG and EGFP tags. The FLAG-EGFP-MASTL was significantly overexpressed (~50×) compared to the endogenous protein (Fig. 2A). Neither the IR-mediated 53BP1 foci formation (Supplemental Fig. S1A) nor the G_2_ arrest (Fig. 2B) was affected by the ectopically expressed MASTL.

These experiments indicate that depletion or overexpression of MASTL does not perturb the rapid DNA damage responses or the subsequent G_2_ cell cycle arrest and DNA repair.

### MASTL controls the timing of mitotic entry after DNA damage

DNA damaged cells can continue the cell cycle and enter mitosis after the DNA is repaired. Alternatively, the cells can enter mitosis even when the DNA is not fully repaired by a process involving adaptation. While cells could repair most of the DNA damage after relatively low dose of IR (2 Gy) (Fig. S1), mitotic entry after higher dose of IR (15 Gy) was mainly due to adaptation (based on the presence of 53BP1 foci at the time of mitotic entry, data not shown). Both cases (as well as forced checkpoint abrogation below) were defined as checkpoint recovery here, which were characterized by mitotic entry and accumulation of histone H3^Ser10^ phosphorylation.

We found that overexpression of MASTL marginally accelerated mitotic entry after DNA damage (Fig. S2A). To overcome the possibility that the expressed MASTL remained inactive, cells expressing a hyperactive mutant of MASTL were generated (changing the lysine at residue 72 to methionine^14^,^15^). Although MASTL^K72M^ was expressed to a level lower than wild type MASTL (Fig. 2C; which is consistent with data from *Drosophila* and *Xenopus*^14^,^15^), it strongly stimulated mitotic entry after DNA damage (Fig. 2D).

Time-lapse imaging provided further evidence that MASTL^K72M^ promoted mitotic entry after DNA damage. While irradiated control cells (grown in the presence of Dox) started to enter mitosis after a delay of 25 hours, MASTL^K72M^-expressing cells entered mitosis significantly earlier, reaching ~50% by 25 hours (Fig. 2E). This was further validated using flow cytometry (Fig. 2F). Both control and MASTL^K72M^-expressing cells were arrested with G_2_/M DNA contents at 20 hours after IR treatment. While the majority of control cells continued to arrest over the next 12 hours, up to half of the MASTL^K72M^-expressing cells recovered and entered G_1_. The different recovery time was not due to different levels of initial DNA damage, because similar number of 53BP1 foci was induced by IR in the presence or absence of MASTL^K72M^ (Fig. S1B). MASTL^K72M^ also did not affect the inactivation of the checkpoint, as CHK2 was inactivated (as indicated by Thr68 dephosphorylation) at the same rate in the presence or absence of MASTL^K72M^ (Fig. 2G).

**Figure 3:**
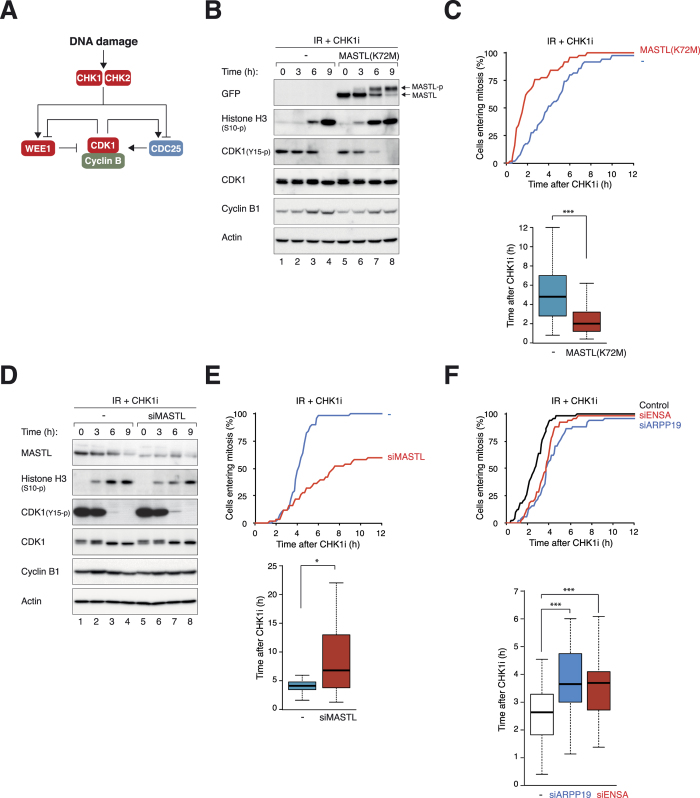
MASTL controls the timing of mitotic entry after DNA damage. (**A**) Inhibition of CHK1/CHK2 bypasses the G_2_ DNA damage checkpoint. DNA damage activates CHK1/CHK2. This results in the activation of WEE1 and inhibition of CDC25, thereby keeping CDK1 in an inhibitory phosphorylated state. Inhibitors of CHK1/CHK2 induce damaged cells to enter mitosis. (**B**) MASTL^K72M^ promotes mitotic entry after AZD7762-mediated checkpoint abrogation. HeLa cells expressing FLAG-EGFP-MASTL^K72M^ were grown in the presence or absence of Dox(48 h) before irradiated (15 Gy). After 16 h, the cells were incubated with AZD7762 (CHK1i) and NOC (to trap cells in mitosis) and harvested at different time points. Phosphorylation of MASTL during mitosis induced a gel mobility shift (MASTL-p). (**C**) MASTL^K72M^ accelerates mitotic entry after checkpoint abrogation. HeLa cells expressing FLAG-EGFP-MASTL^K72M^ were grown with or without Dox(48 h) before transfected with histone H2B-mRFP-expressing plasmids. After 24 h, the cells were irradiated (15 Gy). After another 16 h, the cells were incubated with CHK1i. Individual cells were then tracked with time-lapse microscopy to analyze the time of mitotic entry (*n* = 50)(upper panel). The median mitotic entry time was plotted as a box-and-whisker chart (lower panel). MASTL^K72M^ significantly accelerated mitotic entry (*P *< 0.001). (**D**) Depletion of MASTL delays mitotic entry after DNA damage. HeLa cells were transfected with either control or siMASTL for 48 h before irradiated (15 Gy). After 16 h, the cells were incubated with CHK1i and NOC and harvested at different time points. (**E**) Depletion of MASTL delays mitotic entry after checkpoint abrogation. HeLa cells expressing histone H2B-GFP were transfected with either control or siMASTL for 48 h before irradiated (15 Gy). After another 16 h, the cells were incubated with CHK1i. Individual cells were then analyzed with time-lapse microscopy as in panel (**C**). Depletion of MASTL significantly delayed entry into mitosis (*P *< 0.05). (**F**) Depletion of ARPP19 or ENSA delays mitotic entry after checkpoint abrogation. HeLa cells expressing histone H2B-GFP were transfected with control, siARPP19, or siENSA for 48 h before irradiated (15 Gy). After another 16 h, the cells were incubated with CHK1i and analyzed with time-lapse microscopy as in panel (**C**). Depletion of ARPP19 or ENSA significantly delayed entry into mitosis (*P *< 0.001).

As mitotic entry after irradiation occurred only after a delay of over 24 hours, chemical inhibitors of the checkpoint were also used to mimic checkpoint recovery^16^ (Fig. 3B). As anticipated, incubation of IR-arrested cells with the CHK1 inhibitor AZD7762 promoted mitosis (as detected by histone H3^Ser10^ phosphorylation). This was speeded up after overexpression of either MASTL^K72M^ (Fig. 3B) or MASTL (Fig. S2B). Mitotic entry after AZD7762 treatment was also accelerated by MASTL^K72M^ (Fig. 3C) or MASTL (Fig. S2C). Mechanistically, the accelerated checkpoint recovery was correlated with precocious dephosphorylation of CDK1^Tyr15^, suggesting that MASTL promoted mitotic entry after DNA damage through regulating CDK1 inhibitory phosphorylation (Fig. 3B).

We further performed the converse experiments to determine if silencing of MASTL delayed mitotic entry after DNA damage. Figure 3D shows that the AZD7762-induced histone H3^Ser10^ phosphorylation was delayed after MASTL depletion. Live-cell imaging confirmed that mitotic entry after checkpoint abrogation was delayed after MASTL depletion (Fig. 3E). Similar effects on checkpoint recovery by siMASTL were obtained using another CHK1 inhibitor (UCN-01) (Fig. S3A,B).

ENSA and ARPP19 are two key downstream substrates of MASTL^17^. To test if the effects of siMASTL were specific for the MASTL pathway, we next examined if downregulation of ENSA or ARPP19 could induce similar effects as siMASTL. Immunoblotting showed that ENSA could be depleted with siRNAs (Fig. S4A). As ENSA and ARPP19 share >70% amino acid identity and both proteins were recognized by the antibodies (revealed by using recombinant ENSA and ARPP19, data not shown), our results suggested that ENSA was probably more abundant than ARPP19, and that siARPP19 could further deplete the ENSA/ARPP19 band after ENSA was depleted (Fig. S4A, compare lanes 2 and 6). Although no specific antibodies against ARPP19 were available, siARPP19 could at least effectively downregulate recombinant ARPP19 (Fig. S4B). Moreover, siARPP19 did not affect the level of endogenous or transfected ENSA (Fig. S4A,B).

Consistent with the results using siMASTL, we found that mitotic entry after DNA damage was delayed after silencing of either ARPP19 or ENSA (Fig. 3F). Experiments with siARPP19 and siENSA together were not performed because depletion of both proteins induced substantial cell cycle defects (Fig. S4C) and inhibited entry into mitosis (Fig. S4D).

Collectively, these results indicate that mitotic entry after recovery from the G_2_ DNA damage checkpoint is accelerated by active MASTL and delayed in the absence of MASTL.

### MASTL prevents premature activation of APC/C after DNA damage

**Figure 4:**
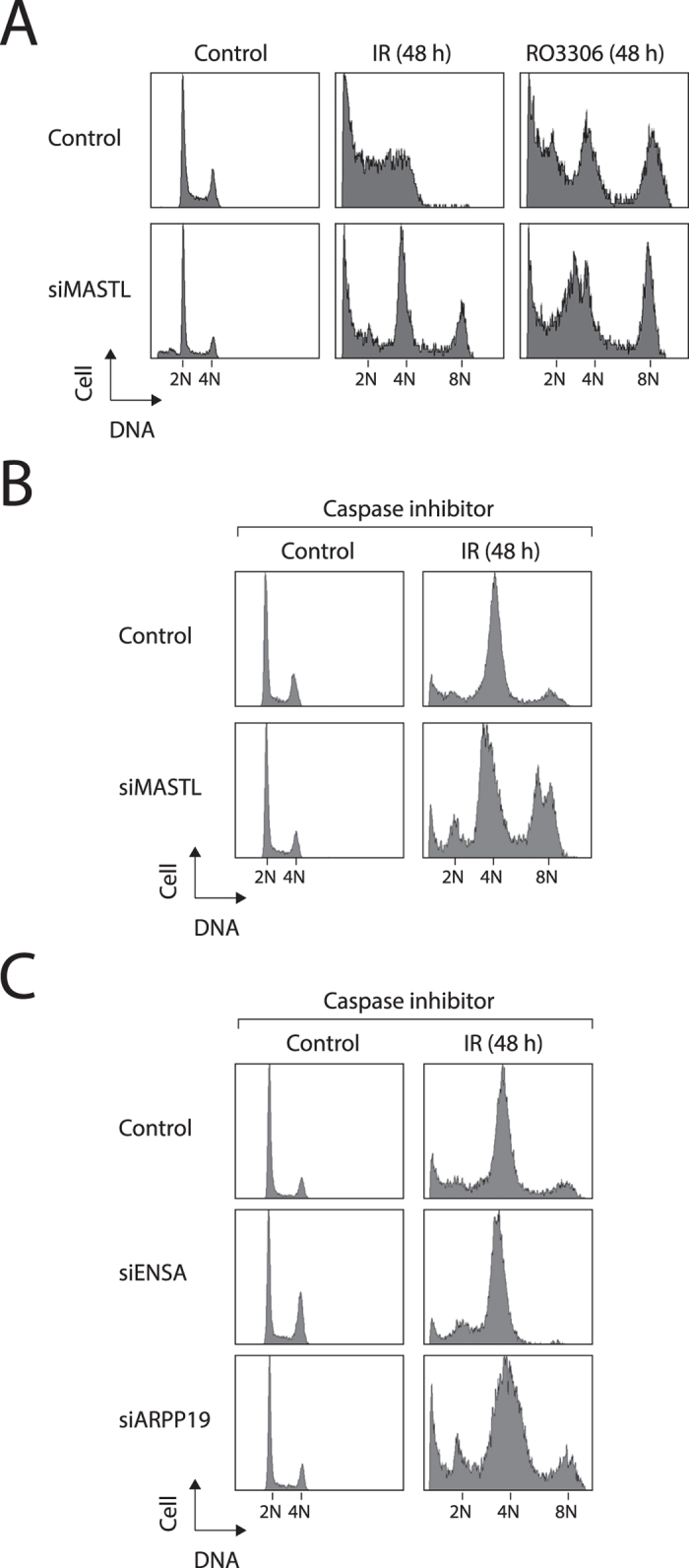
MASTL is required to prevent endoreplication after DNA damage. (**A**) Depletion of MASTL induces DNA re-replication after DNA damage. HeLa cells were transfected with either control or siMASTL for 48 h. The cells were then either irradiated (15 Gy) or incubated with the CDK1 inhibitor RO3306 (CDK1i). After another 48 h, the cells were harvested and analyzed with flow cytometry. The present of cells containing 8N DNA contents indicates endoreplication. (**B**) Depletion of MASTL induces DNA re-replication after DNA damage. HeLa cells were transfected with either control or siMASTL for 48 h. The cells were then irradiated and incubated with a pan-caspase inhibitor. After another 48 h, the cells were harvested and analyzed with flow cytometry. (**C**) Depletion of ARPP19 induces DNA re-replication after DNA damage. HeLa cells were transfected with control, siENSA, or siARPP19 for 48 h. The cells were then irradiated (15 Gy) and incubated with a pan-caspase inhibitor. After another 48 h, the cells were harvested and analyzed with flow cytometry.

We next examined if spontaneous checkpoint recovery is also affected by MASTL depletion. As shown above, MASTL-depleted cells could be arrested in G_2_ at 16 hours after irradiation (Fig. 1F). Further incubation (48 h) resulted in genome re-duplication in siMASTL-transfected but not in control cells (Fig. 4A). By contrast, genome re-duplication induced by pharmacological inhibition of CDK1^18^ occurred in both control and MASTL-depleted cells. Because substantial apoptosis occurred at 48 hours after DNA damage, we also performed the same experiment in the presence of a pan-caspase inhibitor. Figure 4B shows more unambiguously that genome re-duplication occurred in IR-treated siMASTL-transfected cells. These results suggested that in the absence of MASTL, DNA-damaged cells could not sustain a prolonged G_2_ arrest and undergo genome re-duplication. Genome re-duplication of IR-treated cells was also promoted by siARPP19 (but not siENSA), albeit less robustly than siMASTL (Fig. 4C). These results support the hypothesis that the MASTL pathway is required to sustain the DNA damage-mediated G_2_ arrest.

**Figure 5:**
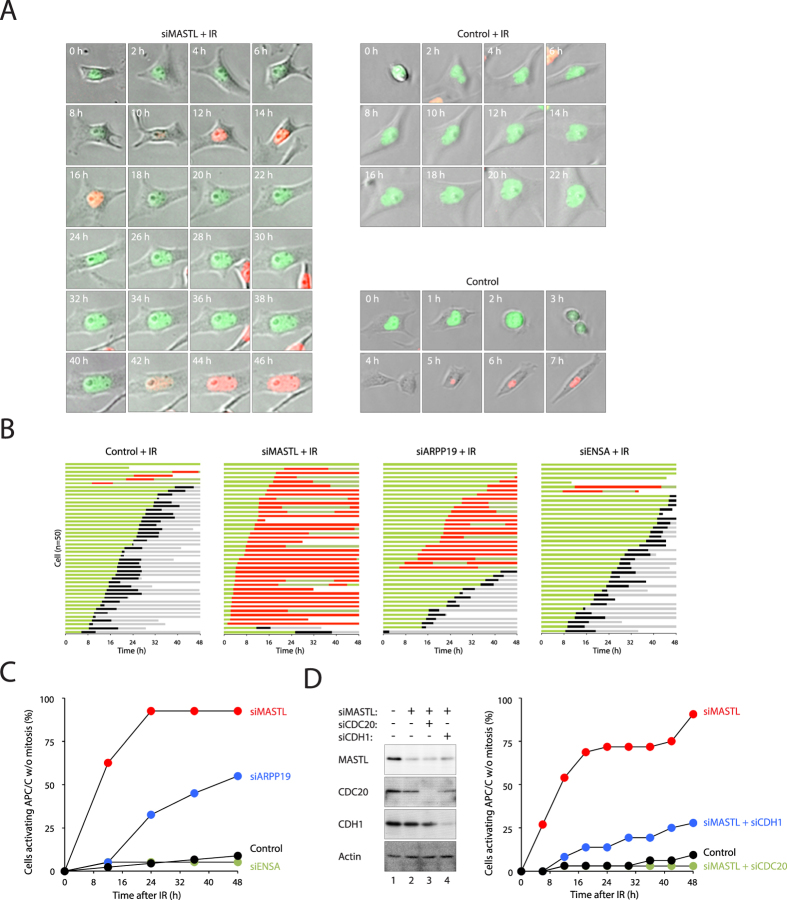
Depletion of MASTL induces premature activation of APC/C after DNA damage. (**A**) MASTL-depleted cells switch from G_2_ to G_1_ directly after DNA damage. HeLa cells expressing FUCCI and BCL-2 were transfected with either control or siMASTL. After 48 h, the cells were treated with IR (15 Gy) and analyzed with time-lapse microscopy. An example of MASTL-depleted cells switching between states that expressed the S/G_2_ reporter (mVenus-Geminin, green) and the G_1_ reporter (mCherry-CDT1, red) without an intervening mitosis is shown (two cycles of oscillation in this example). Examples of control-transfected cells that continued to express the S/G_2_ reporter after IR treatment, and a cell without IR are also shown. (**B**) Depletion of either MASTL or ARPP19 can induce the G_2_–G_1_ switch. HeLa cells expressing FUCCI and BCL-2 were transfected with the indicated siRNAs. After 48 h, the cells were treated with IR (15 Gy). Cells expressing the S/G_2_ reporter at the time of irradiation were then tracked for 48 h with time-lapse microscopy. Each horizontal bar represents one cell (*n* = 50). Green: S/G_2_ (expressing mVenus-Geminin); red: G_1_ (expressing mCherry-CDT1); black: mitosis (from DNA condensation to anaphase); grey: interphase after normal mitosis; truncated bars: cell death. (**C**) Depletion of MASTL or ARPP19 induces premature activation of APC/C after DNA damage. The experiment was performed as described in panel (**B**). The percentages of cells expressing the S/G_2_ reporter that prematurely activated the APC/C without mitosis are quantified (*n* = 50). (**D**) Co-depletion of CDC20 or CDH1 abolishes the premature activation of APC/C in MASTL-depleted cells. HeLa cells expressing FUCCI and BCL-2 were transfected with the indicated siRNAs. After 48 h, the cells were treated with IR (15 Gy). Cells expressing the S/G_2_ reporter at the time of irradiation were then tracked for 48 h with time-lapse microscopy. The percentages of cells expressing the S/G_2_ reporter that prematurely activated the APC/C without mitosis are quantified (*n* = 50). Lysates were also prepared from cells before the imaging and subjected to immunoblotting analysis to verify the depletion of the indicated proteins.

To understand how the cell cycle of MASTL-depleted cells is regulated after DNA damage, cells expressing the FUCCI (Fluorescent Ubiquitin-based Cell Cycle Indicators) cell cycle reporter system were analyzed. The system consists of an APC/C target (mVenus-Geminin, present from early S phase to the end of mitosis) and a SCF complex target (mCherry-CDT1, present from early G_1_ phase to the beginning of S phase)^19^. Instead of using caspase inhibitors to overcome the apoptosis caused by DNA damage, we engineered a cell line to express both FUCCI and the anti-apoptotic protein BCL2. Surprisingly, MASTL-depleted cells did not enter mitosis when they recover from DNA damage. Cells in S/G_2_ (expressing mVenus-Geminin) switched to G_1_ without going through mitosis. Frequently cells underwent multiple rounds of S/G_2_–G_1_ switches within the imaging period (an example is shown in Fig. 5A, Video 1). By contrast, control cells that were in S/G_2_ at the time of irradiation continued to express mVenus-Geminin until checkpoint recovery, when they underwent mitosis (Fig. 5A, Video 2).

We found that while the majority of control irradiated cells eventually entered mitosis, >95% of irradiated MASTL-depleted cells activated APC/C prematurely without mitosis (Fig. 5B,C). Cells that were in G_1_ phase at the time of irradiation behaved similarly, except that they first entered S/G_2_ phase before degrading the APC/C reporter (data not shown). The irradiated MASTL-depleted cells did not display any morphological signs associated with mitosis. This was in contrast to FUCCI-expressing cells that underwent normal mitosis (Fig. 5A, Video 3). Using MASTL-depleted cells expressing histone H2B-GFP instead of FUCCI, we confirmed that there was no DNA condensation to suggest even a very brief mitotic state before APC/C was activated (data not shown).

Similar results were also obtained in U2OS cells expressing the FUCCI reporters (Fig. S5), indicating that the effects of siMASTL on APC/C after DNA damage were not limited to one cell type.

To validate these results, we also used a second siRNA to target MASTL. Although both siRNAs could robustly inhibit the expression of transfected FLAG-MASTL (Fig. S6A), the second siMASTL was less effective in downregulating endogenous MASTL (Fig. S6B). Nevertheless, the second siMASTL also induced prematurely APC/C activation after DNA damage in a large portion of cells (Fig. S6C). We also found that prematurely APC/C activation also occurred after siMASTL-transfected cells were irradiated with lower doses of IR (Fig. S6D).

To further validate the importance of MASTL pathway in preventing premature APC/C activation after DNA damage, we also performed similar experiments using siENSA and siARPP19. Figure 5C shows that siARPP19 (but not siENSA) also promoted premature APC/C activation after DNA damage.

Finally, we verified that the premature degardation of the S/G_2_ reporter in MASTL-depleted cells was indeed APC/C-dependent by targeting critical APC/C components. Cells were transfected with siMASTL either alone or together with siRNAs against CDC20 or CDH1 (two critical targeting subunits of APC/C). The premature activation of APC/C caused by siMASTL was inhibited after co-depletion of either CDC20 or CDH1 (Fig. 5D).

In conclusion, these results indicate that the MASTL pathway is required for DNA-damaged cells to maintain a proper G_2_ arrest; loss of the pathway results in delayed mitotic entry, premature APC/C activation, and endoreplication.

## Discussion

In this study, we provide evidence that MASTL is critical for G_2_ DNA damage responses. Our conclusion is that although activation and inactivation of the DNA damage checkpoint was not affected by MASTL, the speed of mitotic entry after DNA damage and cell fate were determined by the expression of MASTL. While overexpression of MASTL accelerated mitotic entry, downregulation of MASTL delayed mitotic entry after DNA damage. In addition to delaying mitotic entry, downregulation of MASTL also resulted in premature activation of APC/C. Hence the genome instability induced by MASTL downregulation was associated with genome reduplication.

Our available evidence indicated that DNA damage could initially activate the G_2_ DNA damage checkpoint with or without MASTL. For example, 53BP1 foci formation at damaged sites appeared to be normal in the presence of constitutively active MASTL (Fig. S1) or siMASTL (Fig. 1G). The initial G_2_ cell cycle arrest was also independent on MASTL: flow cytometry analysis (Fig. 1F), live-cell imaging (Fig. 1H), and histone H3^Ser10^ phosphorylation (Fig. 1E) all indicated that IR could inhibit mitosis in the absence of MASTL. Similar assays also indicated that IR inhibited mitosis in the presence of constitutively active MASTL (Fig. 2).

Cells irradiated with 15 Gy of IR were able to remain in G_2_ for at least 20 hours before recovery, most likely by adaptation (Fig. 2E). Several methods were employed to measure checkpoint recovery, including the re-accumulation of histone H3^Ser10^ phosphorylation (Fig. 2D), mitotic entry (Fig. 2E), and progression into G_1_ phase (Fig. 2F). They revealed that mitotic entry after DNA damage was accelerated by MASTL^K72M^ (Figs 2D–F and 3A,B) and delayed by siMASTL (Fig. 3D,E). Likewise, mitotic entry following irradiation with a lower dose of IR (2 Gy), which was likely to occur after DNA repair, was also dependent on MASTL (data not shown).

As the kinetics of 53BP1 foci reduction was not affected by MASTL or siMASTL (Figs 1 and S1), it is unlikely that MASTL influenced the rate of DNA repair. Furthermore, mitosis following checkpoint abrogation with a CHK1 inhibitor was also accelerated by MASTL and delayed by siMASTL (Fig. 3), indicating that the effects of MASTL on mitotic entry was independent on DNA repair.

As expected, acceleration of mitotic entry after DNA damage by MASTL^K72M^ was accompanied with an increase of mitotic defects including lagging chromosomes and bridges during anaphase. Cells with more than one observable lagging chromosomes or bridges increased from ~50% in control irradiated cells to ~80% in MASTL^K72M^-expressing cells (data not shown). Moreover, the MASTL^K72M^-expressing cells generally displayed large number of lagging chromosomes and bridges that could not be easily quantified.

The effects of MASTL on checkpoint recovery were probably due to its regulation of the kinetics of CDK1 activation and re-entry into the cell cycle. At least in *Xenopus* egg extracts, MASTL/Greatwall controls inhibitory phosphorylation of CDK1 through CDC25 and WEE1 during G_2_–mitosis^7^. It is possible that human MASTL also regulates CDK1 by promoting the activation of CDC25 and inactivation of WEE1 during checkpoint recovery. In support of this, MASTL^K72M^ did not affect CHK2 inactivation (Fig. 2G) but reduced CDK1^Tyr15^ phosphorylation during checkpoint recovery (Fig. 3B). In contrast, the unperturbed cell cycle of MASTL^K72M^-expressing cells was not significantly shortened compare to that of control cells (data not shown), suggesting that MASTL may be particularly important for cell cycle reentry after DNA damage, when all the cyclin B–CDK1 complexes are in the inhibitory state.

Consistent with the results with MASTL, mitotic entry after DNA damage was delayed after silencing of either ARPP19 or ENSA (Fig. 3F). Depletion of ARPP19 and ENSA together was cytotoxic (Fig. S4). We believe that it was because siARPP19 and siENSA were more efficient than siMASTL. More complete depletion of MASTL is likely to be cytotoxic and unsuited for investigating the DNA damage checkpoint. Development of specific MASTL inhibitors should assist the study of this pathway in the future.

In addition to delaying checkpoint recovery, depletion of MASTL also triggered premature activation of APC/C^CDC20^ during the G_2_ arrest (Fig. 5). This has a decisive effect on genome integrity because degradation of cyclin B triggered G_1_ entry directly, leading to whole genome reduplication (Fig. 4A). MASTL acts on two similar proteins ARPP19 and ENSA. Interestingly, depletion of ARPP19 (but not ENSA) promoted premature APC/C activation (Fig. 5C) and genome rereplication (Fig. 4C) after DNA damage. These results suggest that the MASTL—ARPP19—PP2A pathway may be involved in suppressing APC/C activity after DNA damage. One possibility is that APC/C was spontaneously turned on independently on MASTL after prolonged G_2_ arrest. This does not occur normally because checkpoint recovery occurred before APC/C was activated. When mitotic entry was delayed, as in the case after MASTL depletion, APC/C could be activated independently on MASTL. This is similar to the premature APC/C activation when cells are arrested in G_2_ for a long period of time with the CDK1-specific inhibitor RO3306^18^. However, in contrast to after DNA damage, genome re-duplication induced by pharmacological inhibition of CDK1 occurred in both control and MASTL-depleted cells (Fig. 4A), indicating that the two processes are not identical.

Another possibility is that the MASTL pathway affects APC/C activity directly. However, it is not straightforward to reconcile why depletion of either CDC20 or CDH1 abolished premature APC/C activity. APC/C^CDC20^ is normally activated by cyclin B–CDK1 during mitosis^20^, thereby preparing for the degradation of cyclin B at the end of mitosis. Before that, APC/C^CDC20^ is suppressed by the spindle-assembly checkpoint until all the chromosomes have achieved correct bipolar attachment. By contrast, APC/C^CDH1^ activity is suppressed by CDK1-dependent phosphorylation during mitosis. Hence it is not immediately obvious how a phosphatase pathway could regulate both APC/C^CDC20^ and APC/C^CDH1^ equally.

A protein called EMI1 normally suppresses APC/C activity during G_2_ and is removed before mitosis by a PLK1- and SCF^βTrCP^-dependent degradation. One possibility is that siMASTL induced EMI1 degradation during the IR-mediated arrest, thereby allowing premature APC/C activation. However, we do not favor this idea because EMI1 expression remained unchanged after MASTL was depleted (our published data). Moreover, as EMI1 degradation involves PLK1-dependent phosphorylation, we do not expect siMASTL (which increased PP2A activity) to promote this pathway.

Collectively, these findings highlight the importance of MASTL in maintaining genome stability during DNA damage responses by providing a link between the DNA damage checkpoint and mitotic regulation. On the one hand, it is paramount to keep MASTL inactive during the G_2_ DNA damage checkpoint. Untimely activation of MASTL induced unscheduled mitotic entry (Fig. 2D,E). On the other hand, inadequate activity of MASTL during checkpoint recovery delayed entry into mitosis as well as promoted premature APC/C activation. In this connection, it is intriguing that the level of MASTL differs considerably between different cancer cell lines (our unpublished data). It will be interesting to expand our limited understanding of whether MASTL plays a role in tumorigenesis. Another implication of this study is on the development of MASTL inhibitors, which should be able to delay mitotic entry after DNA damage and sensitize cancer cells to radiotherapies. Indeed, a recent genome-wide siRNA screen revealed that downregulation of MASTL can cause radiosensitization in non-small cell lung cancer cell lines, further supporting MASTL as a promising and druggable target^21^.

## Methods

### DNA constructs

Human MASTL cDNA in pCMV-SPORT6 (IMAGE ID: 3449913) was amplified with PCR using oligonucleotides 5′-CGCCATGGACCCCACCGCGGGAAG-3′ and 5′-CACTCGAGATGAAGGTGTGGGATT-3′; the product was cut with *Nco* I-*Xho* I and ligated into modified pUHD-P3^18^ lacking one *Xho* I site to obtain FLAG-MASTL^C∆310^ in pUHD-P3. The *Xho* I-*Bam*H I fragment from MASTL in pCMV-SPORT6 was ligated into the above MASTL^C∆310^ construct to obtain FLAG-MASTL in pUHD-P3. The *Nco* I-*Bam*H I fragment from FLAG-MASTL^C∆310^ in pUHD-P3 was ligated into pUHD-P3G to obtain FLAG-EGFP-MASTL^C∆310^ in pUHD-P3G. A *Xba* I-*Bam*H I fragment from FLAG-MASTL in pUHD-P3 was ligated into above plasmid to obtain FLAG-EGFP-MASTL in pUHD-P3G. The pUHD-P3G vector was generated as follows: the EGFP fragment from pEGFP-N3 (Clontech, Palo Alto, CA, USA) was amplified with PCR using a vector forward primer and 5′-GGCCCATGGACTTGTACAGCTCGTC-3′; the PCR product was cut with *Nco* I and ligated into pUHD-P3^18^. The *Nco* I site upstream of EGFP was destroyed using Quikchange mutagenesis kit (Stratagene, La Jolla, CA, USA) with the primers 5′-GTTTCAGGGGCCGATGGTGAGCAA-3′ and its complement. A puromycin-resistant cassette was inserted as described into the *Hind* III site to generate pUHD-P3G. Site-directed mutagenesis of MASTL^K72M^ was carried out by Quikchange mutagenesis kit using the oligonucleotides: 5′-TCAACATGAATATGACTCATCAGGT-3′ and 5′-CATATTCATGTTGATCATGTCTGCTT-3′. The *Nco* I-*Xho* I fragment from FLAG-MASTL^C∆310^ in pUHD-P3 was ligated into pGEG-KG to obtain GST-MASTL^C∆310^ in pGEX-KG. FUCCI plasmids (mVenus-Geminin and mCherry-CDT1) were gifts from Atsushi Miyawaki (RIKEN Btain Science Institute, Japan). *Xenopus* 6xHis-ENSA construct was a gift from Satoru Mochida (Kumamoto University, Japan).

### RNA interference

A siRNA targeting MASTL (CCAUUGAGGAAUUCAGCAU, siMASTL#1) was generated by Genepharma (Shanghai, China). A second siRNA against MASTL (GGGACUUGAAACCGGACAA, siMASTL#2) was generated by RiboBio (Guangzhou, China). Two siRNAs targeting ENSA (siENSA#1: UAGCUUUGCCUCUUCAGCUCUCUCA and siENSA#2: GCAGAGCUAGUUGAGAACUCAACAU) and two siRNAs targeting ARPP19 (siARPP19#1: CCUGGAGGUUCAGAUUUCUUAAGGA and siARPP19#2: GAAGAACAAGCAACUUCCUACUGCA) were obtained from Life Technologies (Carlsbad, CA, USA). Unless stated otherwise, siRNA#1 were used in all cases. Cells were transfected with siRNA (15 nM) using Lipofectamine^TM^ RNAiMAX (Life Technologies).

### Cell culture

The HeLa used in this study was a clone that expressed the tTA tetracycline transactivator^22^. U2OS Tet-On cell line was obtained from Clontech (Palo Alto, CA, USA). No authentication was done by the authors. Cells were propagated in Dulbecco’s modified Eagle’s medium (DMEM) supplemented with 10% (v/v) calf serum (HeLa) or fetal bovine serum (U2OS) and 50 U/ml penicillin-streptomycin (Life Technologies) in a humidified incubator at 37 °C in 5% CO_2_.

HeLa cells stably expressing histone H2B-GFP were generated as described previously^23^. HeLa cells expressing FLAG-EGFP-MASTL (or K72M) were generated by transfecting cells with FLAG-EGFP-MASTL (or K72M) in pUHD-P3G followed by selection with puromycin. Single colonies were isolated after about two weeks of selection.

HeLa and U2OS expressing FUCCI were generated by transfection of the FUCCI plasmids followed by cell sorting (FACSAria III, Becton Dickinson, Franklin Lakes, NJ, USA) using 488 nm and 561 nm lasers for excitation. The cells were propagated for one week before being sorted again. Three rounds of sorting were performed. Single cells-derived colonies were obtained by limited dilution of the cells into 96-well plates. The stably expression of BCL2 in the FUCCI/HeLa cells was described previously^24^.

Unless stated specifically, cells were treated with the following reagents at the indicated final concentration: nocodazole (Sigma-Aldrich, St. Louis, MO, USA; 0.1 μg/ml), puromycin (Sigma-Aldrich; 30 μg/ml), AZD7762 (Selleck Chemicals Houston, TX, USA; 20 nM), UCN-01 (Sigma-Aldrich; 100 nM), RO3306 (Santa Cruz Biotechnology, Santa Cruz, CA, USA; 10 μM), Z-VAD-FMK (caspase inhibitor) (Enzo Life Sciences, Farmingdale, NY, USA; 20 μM), doxycycline hydrochloride (Dox) (Sigma-Aldrich; 2 μg/ml). Cell-free extracts were prepared as described previously^25^. Cell cycle synchronization was performed as described^26^. Briefly, G_2_ cells were obtained at 6 h after released from a double thymidine method; mitotic cells were obtained by incubating cells released from a double thymidine method with nocodazole before the mitotic cells were isolated with mechanical shake-off; G_1_ cells were obtained after releasing cells from a nocodazole block.

### Flow cytometry

Flow cytometry analysis after propidium iodide staining was performed as described previously^27^.

### Ionizing radiation

IR was delivered with a caesium-137 source from a MDS Nordion (Ottawa, Canada) Gammacell 1000 Elite Irradiator.

### Live-cell imaging

Cells were seeded onto a 24- or 96-well culture plates and imaged using a Ti-E-PFS inverted fluorescence microscope (Nikon, Tokyo, Japan) equipped with an ultra-low noise sCMOS camera (Andor Technology, Belfast, UK) and a TC temperature, humidity, and CO_2_ control system (Chamlide, Live Cell Instrument, Seoul, Korea). Data acquisition was carried out at 5 min/frame for 24 hours or 10 min/frame for 48 hours. Note that only one of the daughter cells was tracked after mitosis.

### MASTL kinase assay

His-tagged ENSA was prepared as described^2^. Interphase and mitotic MASTL were immunoprecipitated with anti-FLAG agarose from synchronized HeLa cells expressing FLAG-EGFP-MASTL. The immunoprecipitates were then incubated with His-tagged ENSA in the buffer 80 mM Na β-glycerophosphate, 20 mM EGTA, 15 mM MgOAc, 10 mM MnCl_2_, 1 mM DDT pH 7.4 supplemented with 30 μM ATP and 5% v/v^32^ P-γ-ATP at 25 °C for 30 min room temperature. The reaction was stopped by addition of SDS-sample buffer, applied onto SDS-PAGE and analyzed with a PhosphorImager.

### Antibodies and immunological methods

Antibodies against β-actin, FLAG, GFP (Sigma-Aldrich), CDC20, phosphor-histone H3^Ser10^ (Santa Cruz Biotechnology), phosphor-CDK1^Tyr15^, CDC27, CHK2 (BD Transduction Laboratories, Franklin Lakes, NJ, USA), CDH1 (Thermo Fisher Scientific, Waltham, MA, USA), phosphor-CHK2^Thr68^ (Calbiochem, San Diego, CA, USA), and ENSA (also recognize ARPP19) (Abcam, Cambridge, UK) were obtained from the indicated suppliers. Antibodies against CDK1 and cyclin B1 were gifts from Julian Gannon (Cancer Research UK). Rabbit polyclonal antibodies against MASTL were raised against bacterially expressed GST-MASTL^C∆310^ and then purified with a method involving membranes loaded with purified GST-MASTL^C∆310 ^28. Immunoblotting and immunoprecipitation were performed as described previously^25^. For immunostaining of 53BP1, cells were washed twice with ice-cold PBS and fixed with methanol at −20 °C for 10 min followed by blocking with 3% BSA in 0.2% Tween-20 in PBS at 25 °C for 30 min. The cells were then incubated with anti-53BP1 antibody (H-300; Santa Cruz Biotechnology) at 4 °C for overnight. The samples were then incubated with Alexa Fluor 568 conjugated anti-rabbit IgG antibody (Life Technologies) at 25 °C for 2 h. The cells were counterstained with Hoechst 33342 for 1 min and viewed under fluorescence microscope using a 40X objective.

### Statistical analysis

Box-and-whisker plots were generated using RStudio (Boston, MA , USA). Student’s *t*-test was used to calculate statistical significance (*P < 0.05; **P < 0.01; ***P < 0.001).

## Additional Information

**How to cite this article**: Wong, P. Y. *et al*. MASTL(Greatwall) regulates DNA damage responses by coordinating mitotic entry after checkpoint recovery and APC/C activation. *Sci. Rep*. **6**, 22230; doi: 10.1038/srep22230 (2016).

## Supplementary Material

Supplementary Information

Supplementary Video S1

Supplementary Video S2

Supplementary Video S3
