# Supplementary material for: MASTL(Greatwall) regulates DNA damage responses by coordinating mitotic entry after checkpoint recovery and APC/C activation
Source: Sci Rep. 2016 Feb 29;6:22230. doi: 10.1038/srep22230 (PMC4770598; doi:10.1038/srep22230)
Supplement: Supplementary Information [file srep22230-s1.pdf]

MASTL(Greatwall) regulates DNA damage responses by coordinating mitotic entry after checkpoint recovery and APC/C activation

Po Yee Wong, Hoi Tang Ma, Hyun-jung Lee, and Randy Y.C. Poon*

SUPPLEMENTAL FIGURES

SUPPLEMENTAL VIDEO LEGENDS

SUPPLEMENTAL FIGURE LEGENDS

Figure S1. Overexpression of MASTL does not affect DNA damage-mediated 53BP1 foci responses.

(A) DNA damage-induced 53BP1 foci formation is unaffected by MASTL. HeLa cells expressing FLAG-EGFP-MASTL were grown in the absence or presence of Dox for 48 h to turn on or off the recombinant MASTL, respectively. The cells were then treated with IR (2 Gy). The cells were fixed at different time points after irradiation and 53BP1 foci were detected with immunostaining. The number of 53BP1 foci per cell was quantified (*n*=50; mean±95% CI). Examples of 53BP1 staining of MASTL-expressing cells are shown (EGFP signals are also shown).

(B) DNA damage-induced 53BP1 foci formation is unaffected by MASTL^{K72M}. HeLa cells expressing FLAG-EGFP-MASTL^{K72M} were grown in the absence or presence of Dox for 48 h to turn on or off the recombinant MASTL^{K72M} respectively. The cells were then treated with IR (2 Gy). The cells were fixed at different time points after irradiation and 53BP1 foci were detected with immunostaining. The number of 53BP1 foci per cell was quantified (*n*=50; mean±95% CI). Examples of 53BP1 staining of MASTL-expressing cells are shown (EGFP signals are also shown).

Figure S2. MASTL promotes mitosis after checkpoint abrogation.

(A) Overexpression of MASTL speeds up mitotic entry after DNA damage. HeLa cells expressing FLAG-EGFP-MASTL were irradiated (15 Gy). After 16 h, the cells were incubated with NOC and harvested at different time points. The indicated proteins were analyzed with immunoblotting.

(B) MASTL promotes mitotic entry after AZD7762-mediated checkpoint abrogation. HeLa cells expressing FLAG-EGFP-MASTL were grown in the presence or absence of Dox (48 h) before irradiated (15 Gy). After 16 h, the cells were incubated with AZD7762 (CHK1i) and NOC (to trap cells in mitosis). The cells were harvested at different time points and analyzed with immunoblotting. Note that phosphorylation of MASTL during mitosis induced a gel mobility shift (MASTL-p).

(C) MASTL accelerates mitotic entry after checkpoint abrogation. HeLa cells expressing FLAG-EGFP-MASTL were grown in the presence or absence of Dox (48 h) before

transfected with a plasmid expressing histone H2B-mRFP. After 24 h, the cells were treated with IR (15 Gy). After another 16 h, the cells were incubated with CHK1i. Individual cells were then tracked with time-lapse microscopy for 12 h. The time for mitotic entry was analyzed (*n*=50)(upper panel). The median mitotic entry time was also plotted as a box-and-whisker chart (lower panel). Overexpression of MASTL significantly accelerated entry into mitosis ($P<0.05$).

Figure S3. MASTL controls the timing of UCN-01-mediated checkpoint abrogation.
(A) Depletion of MASTL delays UCN-01-mediated checkpoint abrogation. HeLa cells were transfected with either control or siMASTL for 48 h before irradiated (15 Gy). After 16 h, the cells were incubated with UCN-01. NOC was also added to trap cells in mitosis. The cells were harvested at different time points and analyzed with immunoblotting.
(B) Depletion of MASTL delays mitotic entry after checkpoint abrogation. HeLa cells expressing histone H2B-GFP were transfected with either control or siMASTL for 48 h before irradiated (15 Gy). After another 16 h, the cells were incubated with UCN-01. Individual cells were then tracked with time-lapse microscopy for 12 h. The time for mitotic entry was analyzed (*n*=50). The median mitotic entry time was also plotted as a box-and-whisker chart (lower panel). Depletion of MASTL significantly delayed entry into mitosis ($P<0.05$).

Figure S4. Downregulation of ENSA and ARPP19.
(A) Downregulation of ENSA. HeLa cells were transfected with different siENSA and siARPP19 either individually or in combination. After 48 h, lysates were prepared and the indicated proteins were detected with immunoblotting. Equal loading of lysates was confirmed by immunoblotting for actin.
(B) Downregulation of ARPP19. HeLa cells expressing FLAG-ARPP19 were transfected with control, siARPP19, or siENSA. After 48 h, the cells were harvested and analyzed with immunoblotting.

(C) Co-depletion of ENSA and ARPP19 induces a G_2 arrest. HeLa cells were transfected with siENSA and siARPP19 either individually or together. After 48 h, the cells were harvested and analyzed with flow cytometry.

(D) Co-depletion of ENSA and ARPP19 inhibits mitosis. HeLa cells were transfected with siENSA and siARPP19 either individually or together. After 48 h, the cells were analyzed with time-lapse microscopy for 24 h. The percentage of cells entering mitosis during the imaging period was quantified (n=50; mean±SD of three independent experiments).

Figure S5. Depletion of MASTL induces premature activation of APC/C after DNA damage in U2OS cells.

U2OS cells expressing FUCCI cell cycle reporters were transfected with either control or siMASTL for 48 h before irradiated (15 Gy). Individual cells were then tracked with time-lapse microscopy for 48 h. Each horizontal bar represents one cell (n=50). Green: S/G_2 (expressing mVenus-Geminin); red: G_1 (expressing mCherry-CDT1); black: mitosis (from DNA condensation to anaphase); truncated bars: cell death. Note that only one of the daughter cells was tracked after mitosis.

Figure S6. Premature activation of APC/C after DNA damage can be induced by multiple MASTL siRNAs.

(A) Downregulation of MASTL with siRNAs. HeLa cells were transfected with plasmids expressing FLAG-MASTL. After 24 h, the cells were transfected with either control or siMASTL (#1 or #2). After another 48 h, lysates were prepared and the expression of FLAG-MASTL was detected with immunoblotting.

(B) Downregulation of endogenous MASTL. HeLa cells were transfected with either control or siMASTL (#1 or #2). After 48 h, the cells were harvested and analyzed with immunoblotting.

(C) Depletion of MASTL induces premature activation of APC/C after DNA damage. HeLa cells expressing FUCCI and BCL-2 were transfected with either control or siMASTL. After 48 h, the cells were treated with IR (15 Gy). Cells expressing the S/G_2 reporter at the time of irradiation were then tracked for 48 h with time-lapse microscopy.

The percentage of cells that prematurely activated the APC/C without mitosis is quantified (n=50).

(D) Premature activation of APC/C occurs independently on the dose of IR. HeLa cells expressing FUCCI and BCL-2 were transfected with either control or siMASTL. After 48 h, the cells were treated with different doses of IR. Cells expressing the S/G_2 reporter at the time of irradiation were then tracked for 48 h with time-lapse microscopy. The percentage of cells that prematurely activated the APC/C without mitosis is quantified (n=50).

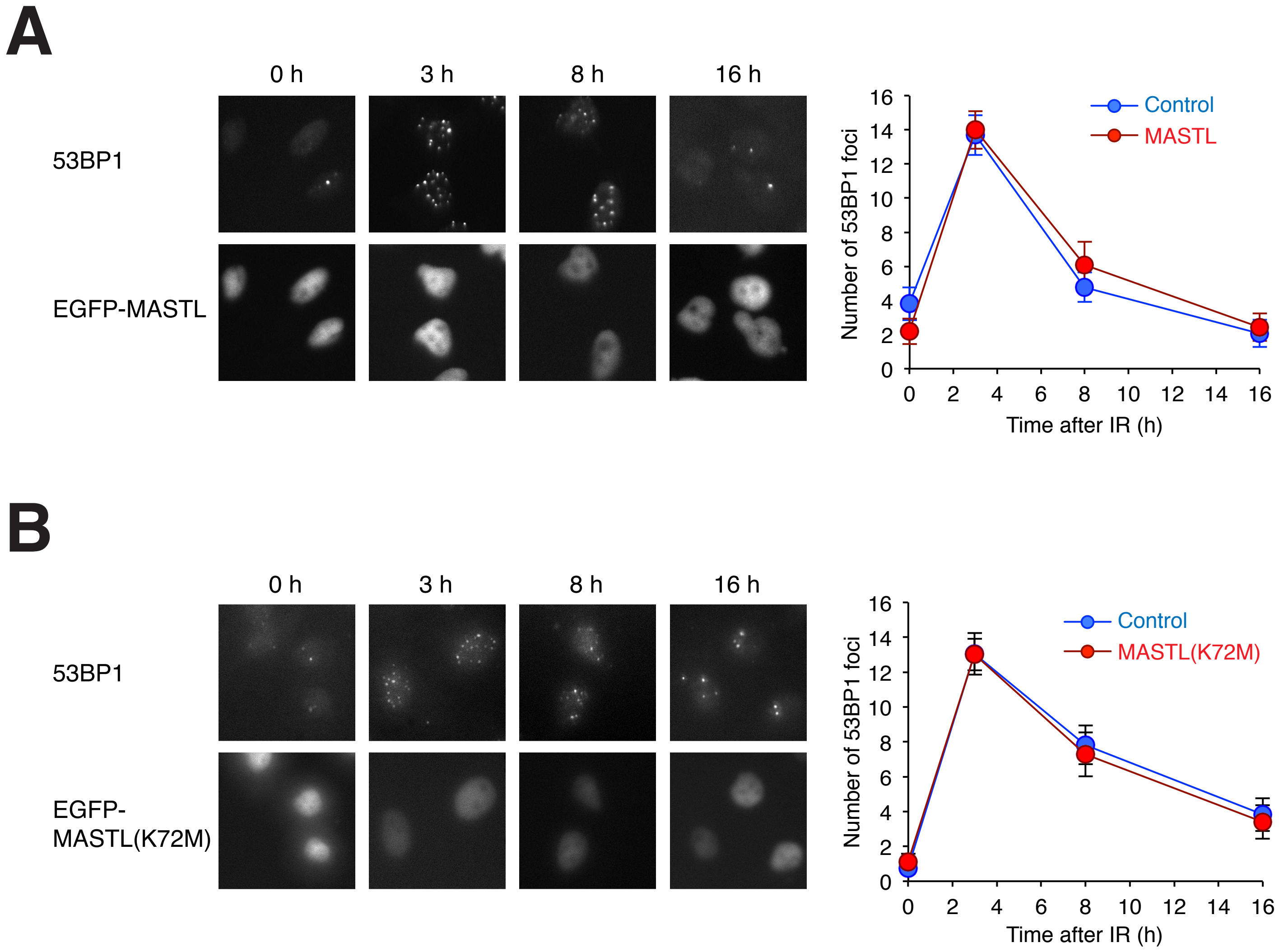

Supplemental Figure S1

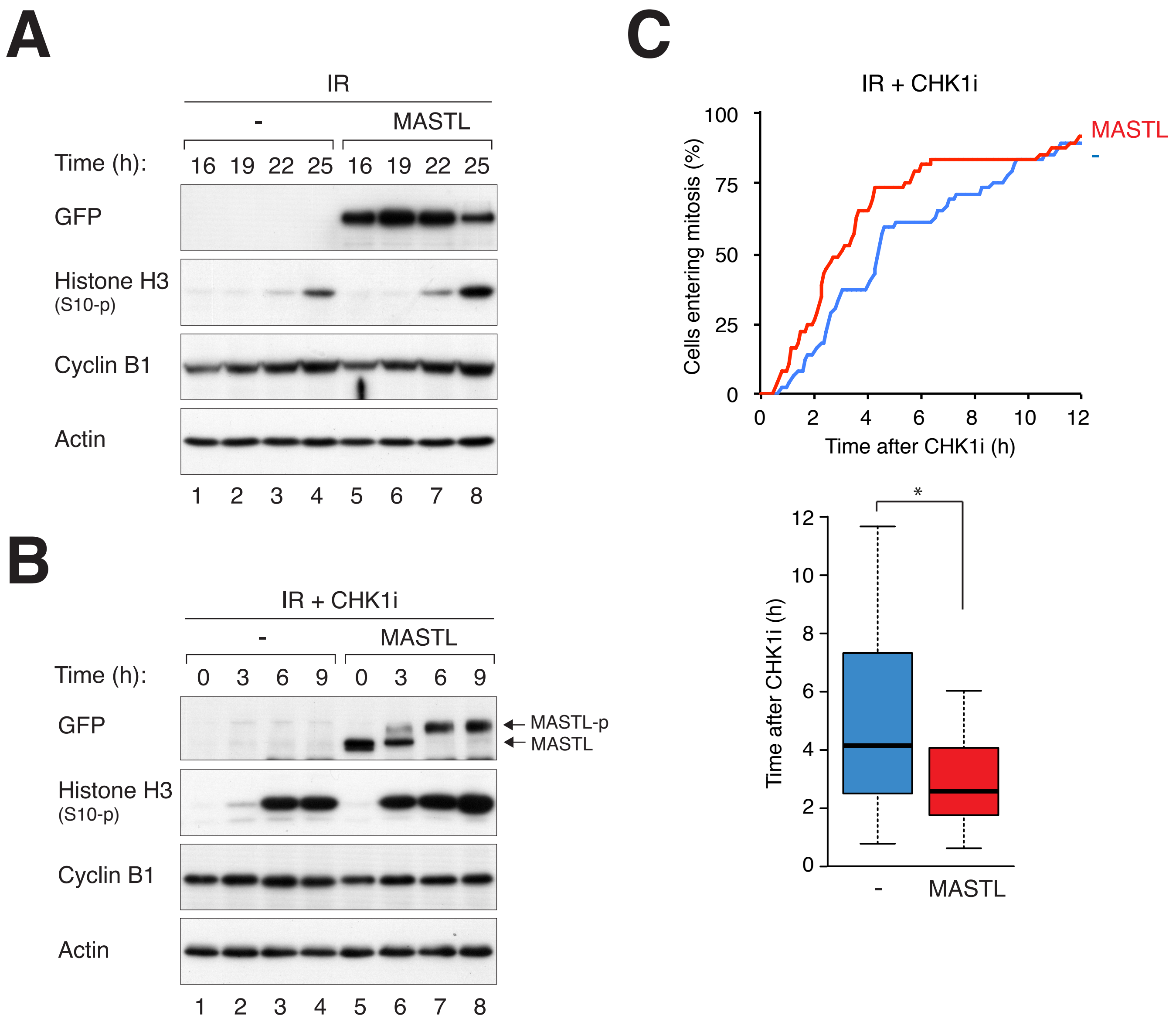

Supplemental Figure S2

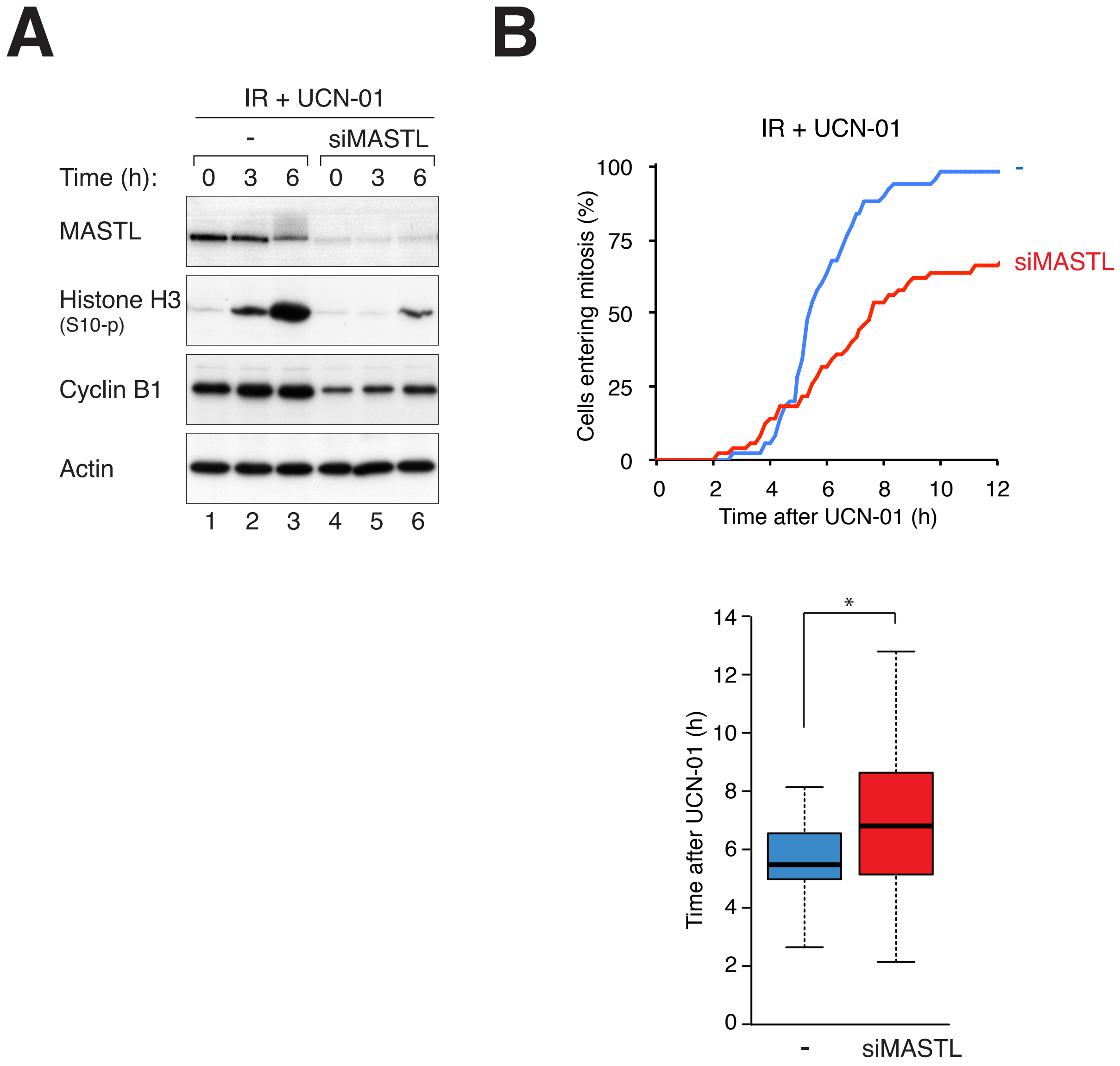

Supplemental Figure S3

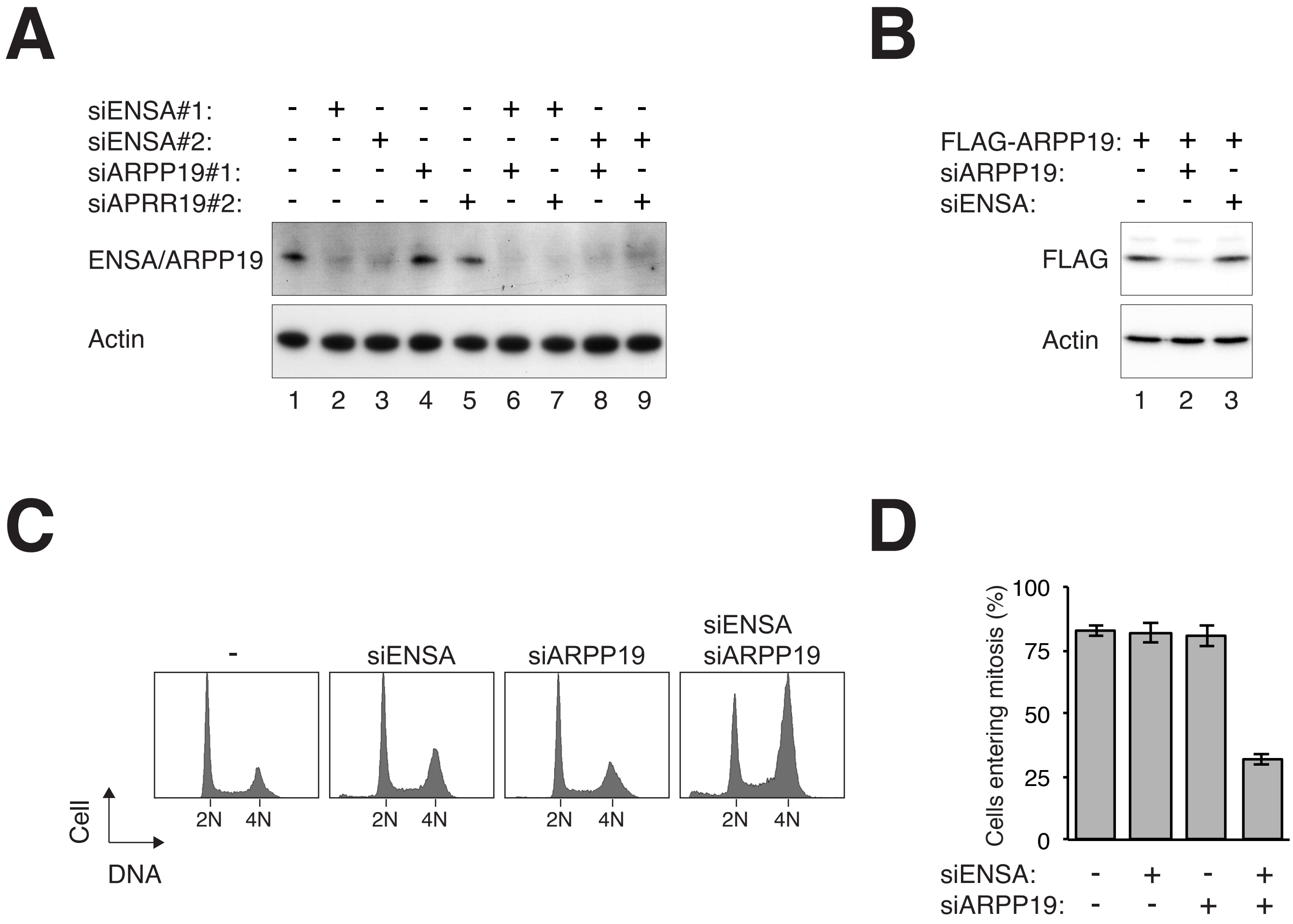

Supplemental Figure S4

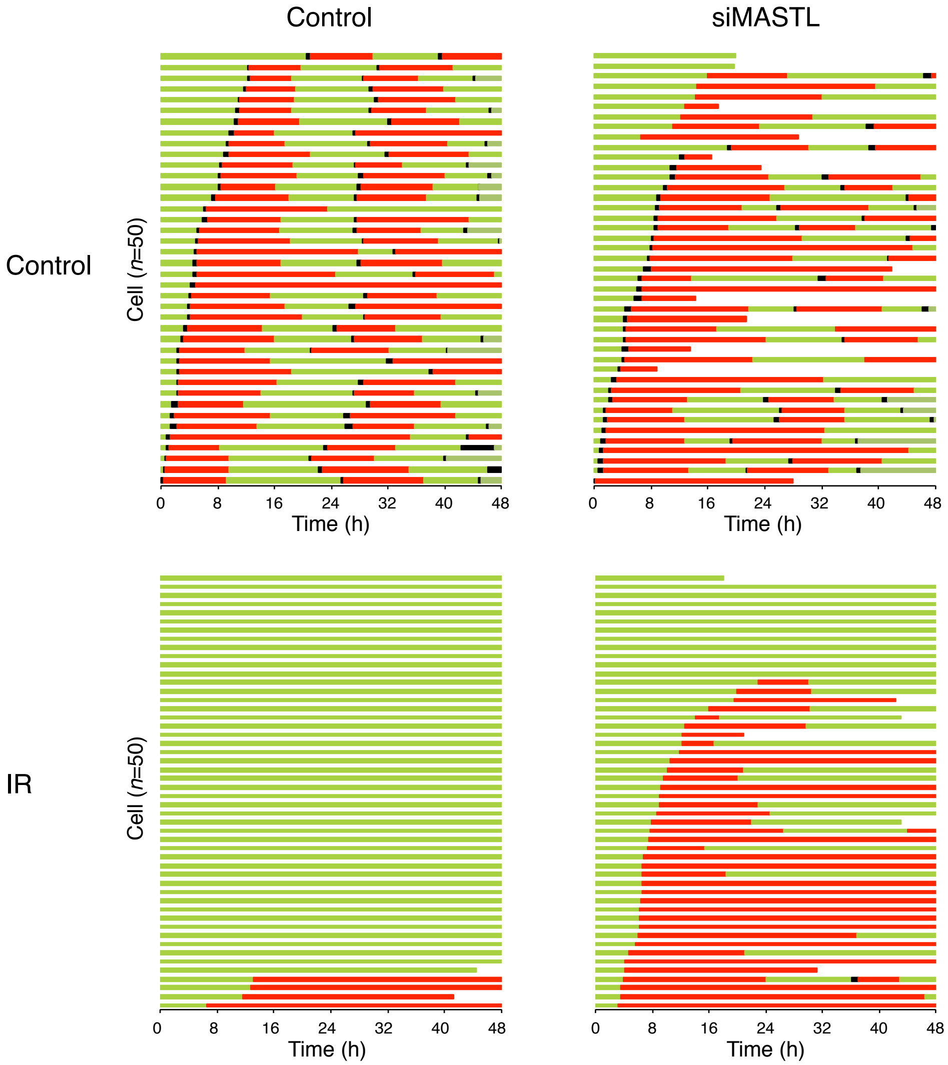

Supplemental Figure S5

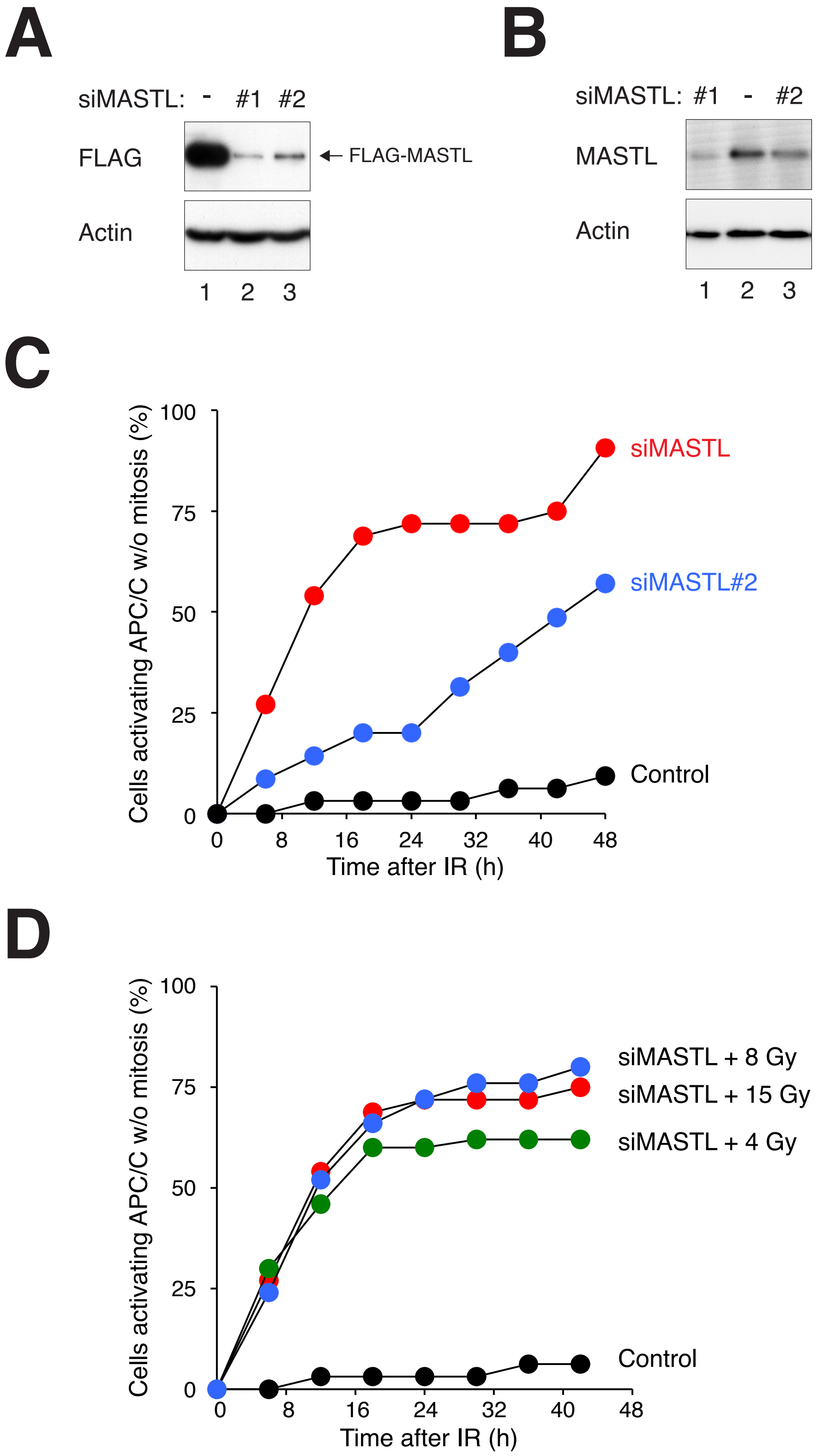

Supplemental Figure S6

VIDEO LEGENDS

Video 1. MASTL-depleted cells switch directly from G_2 to G_1 after DNA damage.

HeLa cells expressing FUCCI and BCL-2 were transfected with siMASTL. After 48 h, the cells were treated with IR and analyzed with time-lapse microscopy. Example of MASTL-depleted cells switching between states that expressed the S/G_2 reporter (mVenus-Geminin, green) or G_1 reporter (mCherry-CDT1, red) without an intervening mitosis is shown (two cycles of oscillation in this example).

Video 2. Control cells are arrested in G_2 after DNA damage.

HeLa cells expressing FUCCI and BCL-2 were mock-transfected. After 48 h, the cells were treated with IR and analyzed with time-lapse microscopy. Example of control cells that continued to express the S/G_2 reporter (mVenus-Geminin, green) after DNA damage is shown.

Video 3. Control cells expressing FUCCI.

HeLa cells expressing FUCCI and BCL-2 were imaged with time-lapse microscopy. Note that cells expressing the G_1 reporter (mCherry-CDT1, red) switched to express the S/G_2 reporter (mVenus-Geminin, green) before destroying the mVenus-Geminin during mitosis.
